# *Saccharomyces cerevisiae*: a versatile eukaryotic system in virology

**DOI:** 10.1186/1475-2859-6-32

**Published:** 2007-10-10

**Authors:** Rui P Galao, Nicoletta Scheller, Isabel Alves-Rodrigues, Tanja Breinig, Andreas Meyerhans, Juana Díez

**Affiliations:** 1 Department of Experimental and Health Sciences, Universitat Pompeu Fabra, 08003 Barcelona, Spain; 2 Institute of Virology, Saarland University, 66421 Homburg, Germany

## Abstract

The yeast *Saccharomyces cerevisiae *is a well-established model system for understanding fundamental cellular processes relevant to higher eukaryotic organisms. Less known is its value for virus research, an area in which *Saccharomyces cerevisiae *has proven to be very fruitful as well. The present review will discuss the main achievements of yeast-based studies in basic and applied virus research. These include the analysis of the function of individual proteins from important pathogenic viruses, the elucidation of key processes in viral replication through the development of systems that allow the replication of higher eukayotic viruses in yeast, and the use of yeast in antiviral drug development and vaccine production.

## Review

The yeast *Saccahromyces cerevisiae *has been successfully used for many years as a model organism to unravel biological processes in higher eukaryotes. Because it is easy to grow and to manipulate genetically it has been always at the forefront of technical advances. For example, *S. cerevisiae *was the first eukayotic organism whose genome was sequenced. The complete yeast genome is known since 1996 and comprises 6000 genes, from which more than 60% have an assigned function. Remarkably, comparative genomic analysis have shown that approximately 40% of yeast genes share conserved amino acid sequences with at least one known or predicted human protein [1]. Moreover, 30% of human genes with a recognized involvement in human diseases have orthologs in yeast [2]. Due to this notable gene homology and the high conservation of fundamental biochemical pathways, studies in yeast have been essential for understanding fundamental cellular processes such as mRNA translation and degradation [3,4], DNA repair mechanisms [5] and the cell cycle [6]. Many of these studies were carried out using classical genetic tools in which a few number of genes can be analyzed at a time. However new technological platforms and tools have been recently created that allow large-scale functional analysis in yeast.

These platforms and tools are commercially available and comprise (i) gene-deletion mutant collections that include ~6000 heterozygous diploid strains, each of which contains a deletion of a single copy of one specific gene, and ~5000 homozygous diploid and haploid strains in which each of the non-essential yeast genes is deleted [7,8], (ii) essential gene mutant collections that include an extensive set of promoter-shutoff strains in which the essential genes are placed under the control of a tetracycline(tet)-repressible promoter [9], and a set of heat inducible shutoff strains to generate ts alleles of essential genes [10]. Both systems allow the study of the ~1000 essential genes in a homozygous background; (iii) gene-expression libraries that include plasmids in which each yeast gene is cloned under the control of the galactose-inducible *GAL1 *promoter. An additional set of plasmids have been created in which the yeast proteins are fused with different tags such as the Flag epitope, the glutathione S. transferase (GST) or the histidine 6 repeat (his6) [11-14]; (iv) DNA microarrays have been created that allow to assess the level of expression of thousands of yeast genes in parallel [15,16], and (v) protein chips containing approximately 5800 yeast proteins fused to GST-his6 [14]. All these tools have opened new avenues in medical research in which systematic genome-wide screenings can be used to characterize disease-related molecular events and to discover novel medical compounds [17-19].

Viruses remain a major thread for human and animal health as well as in agriculture. Nowadays, there is still no vaccine or curative therapy available for main virus pathogens such as the *Human immunodeficiency virus *(HIV) and the *Hepatitis C virus *(HCV). In this respect, a detailed understanding of the biology of pathogenic viruses together with new and systematic screening tools for novel antiviral compounds will be most helpful. As for the cellular biology studies mentioned above, virologist have turned to the use of yeast as a model system to approach fundamental issues in basic and applied research of higher eukaryotic viruses. These studies have exploited the classical yeast genetics and also the recent yeast technological platforms that allow to apply a system biology approach to fundamental questions in virus biology. The present review will discuss first the expression of individual proteins from important pathogenic viruses in yeast to elucidate their function. The vast literature on the yeast two-hybrid system as a method to explore protein-protein interactions however is not a subject of this review. Second, the establishment of yeast/virus systems that allow the replication of higher eukayotic viruses in yeast. These systems have made groundbreaking contributions in the dissection of the life cycle of viruses and the host factors involved. Finally, the third and fourth sections will focus on recent advances in applied virus research, the use of yeast as a tool for antiviral drug development and as a vaccine vehicle, respectively.

### 1. Functional analysis of individual viral proteins in yeast

Although the expression of viral proteins in yeast not always necessarily reflects their role in higher eukaryotes, here we selected some examples in which the analysis in yeast of their effect on highly conserved cellular processes such as cell cycle control, apoptosis or mRNA degradation have contributed to the current understanding of the pathogenesis of important viral pathogens such as *HIV-1 *and *HCV*.

#### Human immunodeficiency virus

With around 40 million people infected worldwide and no effective vaccine or curative therapy, HIV remains a major human thread. A better understanding of the HIV replication cycle and the viral and host factors involved are essential to develop new therapy options. Yeast-based studies have brought to light key aspects of the function of three HIV proteins, the viral protein R (Vpr), the protease (PR) and the regulatory viral protein (Rev). The Vpr protein plays a pivotal role in the pathogenesis of HIV-1. It is involved in the suppression of immune functions and the depletion of infected CD4^+ ^T cells, which finally leads to the progression to AIDS [20]. However, the mechanism underlying its effects on pathogenesis was unknown. Observations initially made in yeast and then corroborated in mammalian cells demonstrated that Vpr acts as an inducer of G2 arrest and CD4^+ ^T cell killing by induction of mitochondrial-dependent apoptosis [21]. The HIV protease is needed to cleave the precursor Pr55^Gag ^to the mature proteins matrix (p17), capsid (p24), nucleocapsid (p7) and p6 proteins [22]. Studies in *S. cerevisiae *demonstrated that the PR specifically arrested cell growth and induced cell lysis mediated by the loss of membrane integrity [23]. The contribution of HIV-1 PR to HIV-induced necrosis was later confirmed with a CD4^+ ^human T cell line, where a PR-specific inhibitor led to prevention of virus-induced cell lysis [24]. These results highlight the importance of PR-inhibitors to prevent CD4^+ ^T cell loss during infection. Finally, the Rev protein plays an essential role in the HIV replication cycle. Rev mediates the export of unspliced and incompletely spliced HIV mRNAs to the cytoplasm and thus, enables the production of HIV-1 particles. The important finding showing that the Rev protein mediates the export of HIV pre-mRNA by interacting with it via cis-acting Rev responsive elements (RRE) in the context of the spliceosome was first shown in yeast [25]. These observations led to the identification in *S. cerevisiae *of the small-nucleoporin-like protein Rip1p as the responsible cellular component for Rev-mediated export [26] and of the Crm1p protein which mediates the interaction between Rip1p and Rev [27].

#### Hepatitis C virus

*HCV *has chronically infected more than 170 million people worldwide and is a major cause of liver cirrhosis and hepatocarcinoma. In spite of the great advances accomplished since its identification in 1989, there are still limited therapy options and no vaccine. The expression in *S. cerevisiae *of two HCV proteins, the non-structural protein 5a (NS5a) and the core protein, together with the construction of HCV chimeras to study HCV RNA translation have made yeast a valuable tool in HCV research. In yeast and human cells, the NS5a protein predominantly associates with the ER membrane through the highly conserved amphiphatic alpha-helix in its N-terminus [28,29]. This is consistent with its proposed role as a key regulator for membrane-associated viral replication. Moreover, studies in yeast and human cells showed that N-terminally truncated NS5a mutants were transported to the nucleus due to a functional NLS and exhibited trans-activating properties [29-32]. Interestingly, the caspase-mediated cleavage of NS5a, occurring during apoptosis or after interaction of NS5a with the core protein during infection, resulted in similar truncations which translocated to the nucleus [33-35]. However, in which way the NS5a-dependent transactivation contributes to HCV pathogenesis still remains to be elucidated.

The HCV core protein interacts with NS5a and is linked to many processes in HCV pathology. From mammalian studies it is known that the HCV core protein influences the transcription of cellular and viral promotors [36], however the mechanisms involved were mainly unknown. Studies in yeast demonstrated the effect of the core protein on the transcription factor AP1-like. The core protein is processed in yeast as in mammalian cells and localizes in the nucleus and the cytoplasm [37-40]. The transport of the mature core protein to the nucleus was shown to be mediated by direct interaction with the ortholog of human importin 4 (Kap123). This interaction in turn abrogates the transport of the AP1-like transcription factor (YAP-1) into the nucleus [37,41] and consequently contributes to the regulation of transcription by the core protein.

A bicistronic vector has been recently designed to study HCV RNA translation in yeast [42]. The HCV RNA is not capped and thus translation depends on an internal ribosomal entry site (IRES) located at the 5'non-translated region (NTR). HCV IRES-mediated translation is modified by the 3'-NTR and several viral and probably also host proteins bind to the 5'-NTR of HCV [43]. Since HCV IRES is functional in yeast [44,45] the bicistronic construct generated will allow to fully explore the effect of viral and cellular factors on HCV translation.

### 2. Replication of higher eukaryotic viruses in yeast

An understanding of the fundamental steps of virus life cycles including virus-host interactions is essential for the design of novel effective antiviral strategies. Such understanding has been hampered by the complexity of higher eukaryotic host organisms. To overcome these experimental difficulties, systems have been developed that allow the replication of higher eukaryotic viruses in *S. cerevisiae*. The first higher eukaryotic virus shown to replicate and encapsidate its genome in *S. cerevisiae *was the *Bromo mosaic virus *(BMV), a positive strand RNA ((+)RNA) virus from plants [46,47]. This pioneer work opened new avenues in virus research and allowed to apply the versatile yeast genetic tools in the elucidation of fundamental steps in virus replication. The list of higher eukaryotic viruses shown to replicate in yeast has been expanded ever since and include (i) other viruses with RNA genomes that infect plants (*Carnation Italian Ringspot virus *(CIRV)*, Tomato Bushy Stunt virus *(TBSV) and *Cymbidium Ringspot virus *(CymRSV)) or animals (*Flock House virus *(FHV) and *Nodamura virus *(NoV)), and (ii) viruses with DNA genomes that infect humans (*Human papillomavirus*), animals (*Bovine papillomavirus*) or plants (*Mungbean Yellow Mosaic India Virus *(MYMIV)) [48-53]. Importantly, these yeast systems reproduce the known features of virus replication in their corresponding natural hosts. Since a detailed description of each of these systems and contributions has been recently reviewed [54,55], we will only highlight some main achievements in virus research. In particular, we will focus on studies carried out with yeast/RNA virus systems since they are the most advanced.

All RNA viruses that have been shown to replicate in yeast belong to the group of (+)RNA viruses. This large virus group includes many important plant, animal and human pathogens such as HCV and *the severe acute respiratory syndrome coronavirus (SARS)*. All (+)RNA viruses share common features in their replication cycles. First, the genomic RNA serves as messenger RNA (mRNA) for translation of the viral proteins and as template for replication. Since these two functions are mutually exclusive, the transfer of the genomic RNA from the cellular translation machinery to the replication complex must be highly regulated. Second, viral replication complexes are associated with intracellular host membranes which proliferate and rearrange in response to the expression of viral protein. And third, host factors are required at multiple steps of the viral replication cycle [56-58].

All the yeast/(+)RNA virus systems developed show common characteristics. (i) the viral RNA-dependent RNA polymerase (RdRp) and additional proteins required for viral replication are expressed in trans from yeast plasmids, (ii) the genomic RNA with authentic 5' and 3' ends is also transcribed from a yeast plasmid or transfected directly into the cell, and (iii) replication is monitored by detecting viral RNA via Northern blot analysis or by measuring the expression of a reporter gene inserted into the viral genome, such that its expression depends on viral RNA replication. The fact that in these systems the individual viral proteins are given in trans from separate plasmids is of great advantage since it allows simplifying and controlling functional studies of each player on viral replication. With this, important aspects of the viral life cycles were studied like translation of viral proteins, the process of template selection, formation of the replication complex, viral RNA replication, encapsidation and recombination (reviewed in detailed in [54,55,59]. These studies have resulted in an impressive progress in the understanding of these fundamental steps of viral RNA replication and the viral proteins involved. Furthermore, due to the common replication strategy of (+)RNA viruses, the results obtained go much beyond the specific virus studied.

The identification of the host factors involved in viral RNA replication is a priority area of research in virology because it can provide new targets for antiviral drug development. The use of traditional yeast genetics and genome-wide screenings have resulted in the groundbreaking identification of multiple host factors required for viral RNA replication and recombination. The gene-deletion mutant collection in which all the non-essential yeast genes are deleted was used to carry out genome-wide screenings of host factors affecting the replication of BMV and TBSV [60,61]. In the BMV studies each of the single deletion strains was transformed with plasmids expressing the BMV replicases (the polymerase 2a and the helicase 1a) and a BMV RNA replication template harbouring a luciferase reporter gene which expression was screened as a marker for BMV RNA replication. In the TBSV studies, a similar approach was applied; however, effects on replication were followed by direct visualization in ethidium-bromide-stained gels of a shorter form of the TBSV RNA, which accumulates at a very high level in the wild-type strain. These analyses revealed that from the approximately 5000 non-essential yeast genes analyzed, around 100 were found to affect the replication of each virus. These results uncovered the involvement in viral replication of previously unconsidered cellular pathways, such as mRNA turnover, stress response and the ubiquitin pathway of protein degradation. Surprisingly and against all previous predictions, only 4 genes were found to be common in both studies. This was discussed to be possibly related to the fact that BMV and TBSV replicate in association with cellular membranes from different compartments.

In spite of the impressive achievement in identifying essential host facors for replication, the above mentioned approach has the limitation that only the non-essential genes can be screened. To overcome this, an additional genome-wide screening was performed in the yeast Tet promoter Hughes collection (yTHC) with TBSV [62]. In the yTHC, which covers 800 of the ~1000 essential genes, each essential gene is under the control of a tet-titratable promoter in the genome [9]. In this way, the expression of the essential gene can be turned off by the addition of doxycycline to the yeast growth medium. With this approach 30 additional cellular genes were identified that are involved in the replication of TBSV [62].

TBSV not only replicates in yeast but also undergoes recombination [49]. To elucidate the eventual role of host factors in the viral RNA recombination process, genome-wide screenings were performed in both yeast collections mentioned above [63,64]. Since the recombinant and non-recombinant TBSV forms vary in length, a screening was performed by direct visualization of TBSV RNA in agarose gels in combination with Northern blot analysis and RT-PCR sequencing. The genetic screens identified 11 nonessential and 16 essential genes that affected TBSV recombination. The exciting observation that host factors can influence viral RNA recombination and thereby evolution has brought a totally new perspective to the field.

### 3. Yeast as a tool for antiviral drug development

Major viral pathogens still remain without effective vaccine or therapy options, thus safer and more effective antiviral drugs are urgently needed. For this, improved screening processes that enable to assess the mode-of-action of the tested compounds are essential. In this section we will discuss the exciting contributions of yeast genetics and high-throughput screenings to drug development. We will present first the principal characteristics of these global approaches and then their application to the antiviral research field.

The process that leads to the discovery and development of new drugs is long and costly. Two approaches have been traditionally used. If the target is already known, the drug discovery search classically starts with *in vitr*o assays to identify small molecule inhibitors. These assays give only a partial view of the effect of the compounds since, for instance, they cannot fully explore drug effects on other additional targets, which can be the source of undesirable side effects. Putative toxic effects will be tested subsequently in cell culture and in animal models. However, if toxicity is detected, the underlying mechanism will still be unknown and no rational approach is possible to modify the drug to improve safety.

An alternative drug discovery approach directly performs cell-based screens for molecules that produce a specific desired effect. This has the advantage that the molecule encounters natural physiological conditions and allows selecting those molecules that are stable within the metabolic environment and discarding those that show toxic effects. However, also with this approach the exact mechanism of action of the selected drug remains unknown. This is a great disadvantage, since its elucidation could lead to the design of new compounds with improved safety and efficacy profiles. Both above approaches will benefit from the large-scale chemical and genetic tools developed in *S. cerevisiae *that allow to systematically screen putative targets and effects of the selected drugs [17,19]. Since the effect of a drug on every yeast gene is analyzed, a broad view of all the proteins and related metabolic pathways affected can be accomplished. Thus, including yeast-based screenings at an early stage in drug development programmes, crucial information will be rapidly obtained that can be used to discard or to improve the tested compounds.

The large-scale chemical and genetic yeast screens use the mutant strain collections and gene expression plasmids described above. There are five main screening approaches [19]. First, the drug-induced haploinsufficiency approach uses the heterozygous mutant collection and is based on the finding that reducing the copy number of a drug target gene from two to one copy can significantly sensitize a diploid cell to that drug [65-67]. The second approach uses the haploid or the homozygous diploid deletion collection and is based on the principle that the deletion of a gene that renders cell-hypersensitivity to a specific drug identifies pathways that buffer the cell against the toxic effect of the drug and thus provides clues about its mode of action [68,69]. The third approach uses gene expression libraries and is based on the principle that increasing the concentration of a protein that is the target of an inhibitory drug should increase resistance to the drug. These three approaches can be performed in two different ways, (i) by growth measurement of arrayed strains, and (ii) by competitively growing pooled strains. The fourth approach is based on gene expression profiling and consists of comparing changes in mRNA expression elicited by a drug of interest with those induced by other drugs or by gene deletions. Finally, the plasmid collection of all yeast proteins expressed as GST-fusions was used to create protein chips that allow the screening of direct drug-protein interactions. The combination of all the five powerful techniques together with the rapidly growing knowledge of yeast genetic networks[70] has made yeast a recognized system in drug development that until today has not been fully exploited in antiviral research.

After more than two decades of searching for antiviral drugs, a rather limited number of compounds is on the market. From these, almost all focus on HIV and *Herpesviruses*. Furthermore, most antivirals are not curative and produce major side effects. Thus, there is an urgent need of novel and high-quality targets and drugs together with improved screening systems to identify them. Two kinds of targets can be used in antiviral research, viral factors or host factors. Classically, viral proteins have been used as targets for therapeutic interventions because the developed inhibitor molecules were expected not to produce significant side effects. However, this does not seem to be the case and, for instance, all the antiretrovirals currently available produce important side effects. Since drug-resistant mutants are rather easily selected, researchers have shown again interest in host factors. They have the advantage of being genetically stable and may even be efficient as targets against groups of viruses. The above mentioned screening options could be helpful to select those host factors that when targeted by drugs will be lethal for the virus but have minor effects on the cell.

Two yeast cell-based assays have been established to screen for viral inhibitors. The protein M2 of *influenza virus *has a fundamental role in the disassembly of the influenza virion. In fact, some of the commercially available drugs target this protein.

Since expression of the M2 protein in yeast produced a slower growth rate, a screening procedure was set up based on the rescue of wt cell growth in the presence of test compounds [71]. More recently, a parallel approach has been applied to the serine protease encoded by the human cytomegalovirus (HCMV) [72]. This protease is essential for the virus and recognizes a 40 amino acid sequence, which was inserted into the coding sequence of a yeast enzyme involved in the biosynthesis of tryptophan. Coexpression of the HCVM protease with the engineered enzyme resulted in an arrest of cell growth in media without tryptophan that could be rescued in the presence of protease inhibitors. This system provides the basis for the high-throughput screening of inhibitory molecules. Related to the analysis of host factors as antiviral targets, the genome-wide screenings in the yeast/virus systems described in the previous section have provided many new candidates for which the therapeutic potential remains to be explored.

Few studies in antiviral research make fully use of the yeast platforms and tools available. Because of their innovation in the field, we would like to point out two of them that explore the mode of action of antivirals plus other therapeutic compounds. In the first study the heterozygote yeast deletion collection was used to asses the cellular effects of 78 different compounds [67]. It is important to mention that in the construction of this collection, each yeast gene was replaced with a cassette containing the selectable marker gene *KanMx *plus two unique tag sequences located up- and down-stream of the marker gene. These tag sequences enabled rapid analysis of strains in a pool by using hybridization to high-density nucleotide arrays. Importantly, the tag sequences were flanked with universal primer sets for polymerase chain reaction amplification [8]. Thus, a pool of ~3500 heterozygous deletion strains were competitively grown for 20 generation in media containing the selected compounds. Then, DNA was extracted from the cell culture at generation 0 and 20 and amplified by PCR using universal primers and Cy3 (green) or Cy5 (red) labels, respectively. The amplified DNA was hybridized to microarrays containing each specific tag sequence. Since signal intensities from microarray hybridizations are proportional to the relative tag abundance in the pool population, for each yeast strains red, green or yellow colours in the array will correlate with an enrichment, depletion or unchanged representation of the strain in the population. In this way the effect of a drug on each of the yeast deletion strains could be rapidly analyzed [67].

In a second study a complementary approach was followed. The ~5000 yeast haploid deletion mutant collection was tested for hypersensitivity to 82 different compounds including some molecules with antiviral activity [69]. Since it is considered that compounds with similar biological effects lead to similar chemical profiles, clustering of the obtained results provided a very useful data set that allowed the organization of the compounds into functionally relevant groups. The results obtained with both studies helped to identify new modes of action of the tested compounds and revealed numerous insights into the cellular pathways involved,

### 4. Yeast as a vaccine vehicle

Yeast has been utilized in vaccine development. The classical example is the recombinantly expressed hepatitis B surface antigen that has become a safe and efficient prophylactic vaccine worldwide [73]. However, in this section we will focus on its use not as a mere production tool for recombinant antigens but as a vaccine vehicle itself.

Immunization against bacterial pathogens and viruses is an attractive strategy in combating infectious diseases by stimulation of the immune system. Traditional vaccines like soluble antigens formulated with adjuvants efficiently activate the humoral immune response. The production of neutralizing antibodies that bind to invading microorganisms inhibits their entry into target cells, thereby preventing an infection. However, cell-mediated immunity, not humoral immunity alone, is required to control infections with intracellular pathogens, especially viruses like HIV and HCV. In the classical view of cellular immunity, antigen-presenting cells (APC) present peptides from endogenously synthesized pathogen proteins in the context with MHC class I molecules on their cell surface. Antigen-specific CD8-positive T lymphocytes become activated by recognizing these peptides and subsequently differentiate into a mature effector cell population. These cytotoxic T cells (CTL) are able to recognize and kill the respective infected cells. Additionally, dendritic cells (DC) which are the most potent APC use the mechanism of cross-priming for antigen presentation, wherein peptides from exogenous antigens are also presented via MHC class I molecules [74]. "Danger" signals as given by the interaction of pathogen components (e.g. cell wall components of yeast) with pattern recognition receptors on the surface of APC, such as Toll-like receptors (TLR), mannose or glucan receptors, leading to the maturation of the APC and an efficient antigen presentation [75,76].

Whole recombinant yeast cells, known as Tarmogens™ (targeted molecular immunogens) represent a novel vaccination tool for the induction of potent cell-mediated immune responses against target antigens [77]. In most cases, the non-pathogenic baker's yeast *Saccharomyces cerevisiae *has been used as antigen carrier because this genus combines several advantages. (1) Products with *S. cerevisiae*, e.g. bread and beer, are consumed in large amounts worldwide and healthy individuals show only moderate T cell responses against *S. cerevisiae*, probably due to mechanisms of oral tolerance [78]. (2) Important for prime-boost immunization strategies is the observation that there is no neutralization of yeast vectors by the host immune system following multiple immunizations in mice and rabbits [79,80]. Remarkable low titres of anti-yeast antibodies were found after repeated injections of either live or heat-killed antigen-expressing *S. cerevisiae *[77]. (3) Yeast cells themselves have potent adjuvant effect via "danger" signals that lead to the maturation of DC without being dangerous, so no exogenous adjuvant is needed [80,81]. (4) Facilitated by the particulate structure of yeast, antigens are efficiently presented via the MHC class I pathway resulting in the activation of antigen-specific CD8 T lymphocytes [81-83].

Stubbs et al. showed for the first time that recombinant yeasts expressing HIV-1 antigens and tumour antigens potently elicit antigen-specific CTL responses, including those mediating tumour protections in mice [80]. Further studies with the animal tumour challenge model could demonstrate that yeast-based immunotherapy using mutated Ras oncoprotein as target antigen caused a mutant-selective tumour remission in animals [77,79]. This concept is now being tested in a Phase I clinical trial in patients with colorectal, pancreatic and non-small cell lung cancers [77]. Most recently, the same group has evaluated a recombinant yeast cell vaccine that expresses the HCV NS3-Core fusion protein as an immunotherapeutic vaccine in chronic HCV-infected individuals [84].

One advantage of using whole recombinant yeasts as antigen carriers is the oral application route. Feeding mice with *S. cerevisiae *expressing *Actinobacillus pleuropneumoniae *antigen led to the induction of protective systemic and mucosal immune responses [85]. The fission yeast *Schizosaccharomyces pombe *represents, like *S. cerevisiae*, a physiologically and genetically well characterized eukaryote that has been used in Africa for hundreds of years in brewing of millet beer, from which it was first isolated. Recombinant *Sz. pombe *cells expressing the human cytomegalovirus pp65 protein were able to stimulate CD4- and CD8-positive memory T cells in human blood [82].

Instead of using recombinant yeast expressing an antigen of choice as a vaccine, attempts have been made to express MHC proteins plus the desired antigenic peptide on the yeast surface and to use the modified cell as a kind of APC. Brophy and colleagues succeeded in expressing mouse MHC I protein heavy chain, beta2-microglobulin plus an antigenic peptide from a single gene cassette. The resulting complex assembled in a functional conformation on the cell surface and even induced the activation of naïve T cells [86]. However whether this approach will be of practical use in vaccination has yet to be demonstrated.

The strategy to use yeast cells for vaccination could also be used to elicit protective immune responses against human pathogenic yeasts. For example, the genus *Candida *is able to cause a variety of infections from mucosal candidiasis to life-threatening disseminated and bloodstream infections, especially in immunocompromised individuals. Unfortunately, the interplay between protective and inhibitory antibodies dictates the outcome of experimentally disseminated Candidiasis in mice receiving a heat-inactivated whole-cell *C. albicans *vaccine [87]. This observation may explain why subjects with elevated anti-*Candida *antibody titers remain nonetheless susceptible to invasive candidiasis [87]. However, Ibrahim and colleagues could show that vaccination with the recombinant N-terminal domain of the adhesin Als1p improves survival during murine disseminated candidiasis by enhancing cell-mediated, not humoral, immunity in both, immunocompetent and immunocompromised mice [88,89].

## Conclusion

In conclusion, the yeast system has been extremely valuable in virus research. However, the full potential from the remarkable progress in systemic yeast biology over the last years has yet to be exploited. With the increasing options for analysing protein interaction networks at an unprecedented level of complexity and the rising number of viral replication systems in yeast that correctly mimic the fundamental properties of the virus life cycle in higher eukaryots, the era of system virology is expected to become a new focus in virus research. Subsequently, the better understanding of the virus protein/host cell protein interaction networks will allow the search for and development of more efficient and safer antiviral drugs. Moreover, the acquired information about drug functioning down to the molecular level will help in the search for new drug applications and might significantly reduce the time frame for drug development when a new virus appears to threaten the human population.

## Competing interests

The author(s) declare that they have no competing interests.

## Authors' contributions

All authors have contributed to the content and structure of the present review.
